# The implementation of the acute care surgery model in the management of patients with acute appendicitis – A 5-year single-center, retrospective experience: An observational study

**DOI:** 10.1097/MD.0000000000038927

**Published:** 2024-07-19

**Authors:** Kyoung Won Yoon, Keesang Yoo, Kyoungjin Choi, Eunmi Gil, Chi-Min Park, Donghyoun Lee

**Affiliations:** a Division of Critical Care, Department of Surgery, Chung-Ang University Gwangmyeong Hospital, Gwangmyeong, Korea; b Division of Acute Care Surgery, Department of Surgery, Samsung Medical Center, Sungkyunkwan University School of Medicine, Seoul, Korea; c Department of Critical Care Medicine, Samsung Medical Center, Sungkyunkwan University School of Medicine, Seoul, Korea; d Department of Surgery, Jeju National University Hospital, Jeju National University School of Medicine, Jeju, Korea.

**Keywords:** acute care surgery, appendicitis, clinical decision-making, emergency service, hospital, length of stay

## Abstract

We conducted this study to assess the effects of the acute care surgery (ACS) model in the management of patients with acute appendicitis (AA) based on our 5-year single-center, retrospective experience. The current single-center, retrospective, observational study was conducted in a consecutive series of the patients with AA who had been surgically treated at a tertiary referral hospital in Seoul, Korea, between January 2016 and December 2020. At our institution, the ACS model was first introduced in March 2018. Therefore, our clinical series of the patients were divided into 2 groups: the pre-ACS group (March 2014 to February 2018) and the post-ACS group (March 2018 to December 2022). Key time intervals include emergency department registration to request for surgical consultation, request for surgical consultation to decision on surgery, decision-to-operating room, time to decision on surgery and length of emergency department stay. Moreover, outcomes include rates of perforation and complications and discharge within 24 or 48 hours. We compared key time intervals, outcomes, and length of hospital stay between the 2 groups. A total of 900 patients with AA were finally included in the current study, 447 and 453 of whom were divided into the pre-ACS group (n = 447) and the post-ACS group (n = 453), respectively. There were significant differences in key time intervals, outcomes, and length of hospital stay between the 2 groups (*P* < .05). In conclusion, our results showed that the implementation of the ACS model was effective in improving key time intervals, rates of perforation, and discharge within 24 or 48 hours in the patients with AA.

## 1. Introduction

Appendectomy is one of the most common surgeries. Any delays in surgery in patients with acute appendicitis (AA) would raise a risk of perforations and complications.^[1]^ AA is the most common abdominal surgical emergency worldwide, with an annual incidence of 96.5 to 100 cases/100,000 adults.^[2]^ To date, medical treatment has been proposed as an alternative to surgery in patients with AA. Still, however, surgery remains a mainstay of treatment in a certain type of patients, such as those with an appendicolith.^[3,4]^

Over the past decade, as surgical subspecialization has progressed, the number of general surgeons capable of performing elective surgery, nontrauma emergency surgery, and trauma surgery has steadily decreased. In response to these changes, the concept of acute care surgery (ACS) emerged and has been discussed since the early 2000s in various countries in the United States and Europe, specializing in emergency surgery and trauma patient management.^[5,6]^ Depending on the socioeconomic conditions and surgical training processes of each country, ACS has developed in slightly different forms. However, it is generally accepted as a field that encompasses emergency surgery, trauma surgery, and the management of critically ill surgical patients.^[5–9]^ In the United States, the American Association for the Surgery of Trauma (AAST) plays a central role in ACS, and the ACS Committee was established under its umbrella. The ACS Fellowship Program was initiated in 2008, and currently, ACS programs are being implemented in 21 institutions.^[10]^ Several studies have been published on ACS programs, and a recent systematic review reported that the ACS model reduces mortality rates, complication rates, surgical time, and financial costs compared to the traditional surgical model.^[11]^ In a recent nationwide study in the United States, it was reported that certified ACS programs operating in Level 1 trauma centers resulted in shorter hospital stays, lower complication rates, and lower overall hospital costs for patients receiving emergency surgical care compared to trauma centers or nontrauma centers without such programs.^[12]^ According to Knowlton et al,^[13]^ the ACS system was responsible for increased rates of health care use, a prolonged length of hospital stay (LHS) and increased cost.

Given the above background, we conducted this study to assess the effects of the ACS model in the management of patients with AA based on our 5-year single-center, retrospective experience.

## 2. Materials and methods

### 2.1. Study patients and setting

The current single-center, retrospective, observational study was conducted in a consecutive series of the patients with AA who had been surgically treated at a tertiary referral hospital (Samsung Medical Center, Sungkyunkwan University School of Medicine) in Seoul, Korea between January 2016 and December 2020. It was approved by the Institutional Review Board (IRB approval #: 2020-09-178) and a written informed consent was waived due to the non-interventional nature of the study design.

Inclusion criteria for the current study are as follows:

1. Korean adults aged > 18 years old.
2. The patients with a confirmed diagnosis of AA at the emergency department (ED).
3. The patients undergoing surgery.

Exclusion criteria for the current study are as follows:

1. The patients lost to follow-up.
2. The patients aged 16 years or younger.
3. The patients with septic shock who underwent exploratory laparotomy.

### 2.2. The concept and implementation of the ACS model

Before the implementation of the ACS model, a mixed group of physicians, such as professors, fellows and residents from other specialty areas, as well as duty residents of department of general surgery were primarily responsible for assessing patients complaining of abdominal emergencies in the ED. This was followed by decision-making supervised by on-call doctors according to the resident’s presentation over the telephone. It was therefore difficult to ensure the quality of surgical outcomes. After the implementation of the ACS model, however, in-house attending trauma surgeons were primarily responsible for assessing and treating patients with nontrauma abdominal surgical emergencies.^[14–16]^

**Figure 1.**
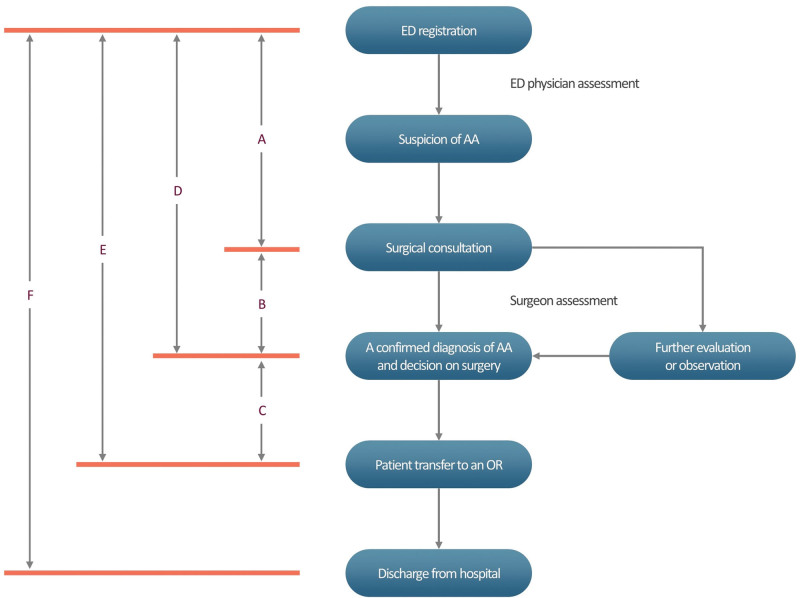
The concept and implementation of the acute care surgery model.

At our institution, a diagnosis of AA is commonly made based on patients’ clinical presentation or enhanced computed tomography (CT) scans, as previously described.^[17]^ A CT imaging is available 24 hours a day and can be performed within an hour after in-house attending trauma surgeons decide to perform it. The concept and implementation of the ACS model is illustrated in Figure 1.

### 2.3. Patient evaluation and criteria

At our institution, the ACS model was first introduced in March 2018. Therefore, our clinical series of the patients were divided into 2 groups: the pre-ACS group (March 2014 to February 2018) and the post-ACS group (March 2018 to December 2022).

Baseline characteristics of the patients include age, sex, body mass index (kg/m^2^), underlying diseases, type of surgery, conversion to open surgery, systolic blood pressure, body temperature, white blood cells, C-reactive protein, and lactic acid.

Key time intervals (min) include ED registration to request for surgical consultation, request for surgical consultation to decision on surgery, decision-to-operating room (OR), time to decision on surgery and length of ED stay, as defined in Figure 1.

Outcomes include rates of perforation and complications and discharge within 24 or 48 hours.

LHS is also defined in Figure 1.

### 2.4. Statistical analysis of the patient data

All statistical analyses were performed using the IBM SPSS Version 27.0 for Windows (IBM Corp., Armonk, NY) and a *P* value of <.05 was considered statistically significant. Values were expressed as mean ± standard deviation (SD) or the number of the patients with percentage, where appropriate. To analyze differences in them between the 2 groups, we used Student *t* test, or Mann–Whitney *U* test, Pearson χ^2^-test or Fisher exact test, where applicable.

## 3. Results

### 3.1. Baseline characteristics of the patients

The current study was conducted at a tertiary hospital equipped with a total of 16 operating rooms including 7 for emergency surgeries, if necessary, where approximately 140 patients are transferred from other hospitals the remaining 60 walk in every year. Its ACS division was staffed by 5 surgeon-intensivists who are dual-certified by the Korean Society of Surgery and the Korean Society of Critical Care Medicine. Of these, 2 were predominantly tasked with intensive care unit (ICU) responsibilities and the remaining 3 focused on emergency surgeries on a rotating basis. Because the ACS division does not have its own operating room, similar efforts should be made to secure an operating room before and after the implementation of the ACS system.

The occupancy rate for the general surgical ICU exceeded 85% on weekdays and 70% on weekends. Between 2016 and 2019, approximately 300 and 80 emergency surgeries were annually performed at the emergency department and for inpatients, respectively.

**Figure 2.**
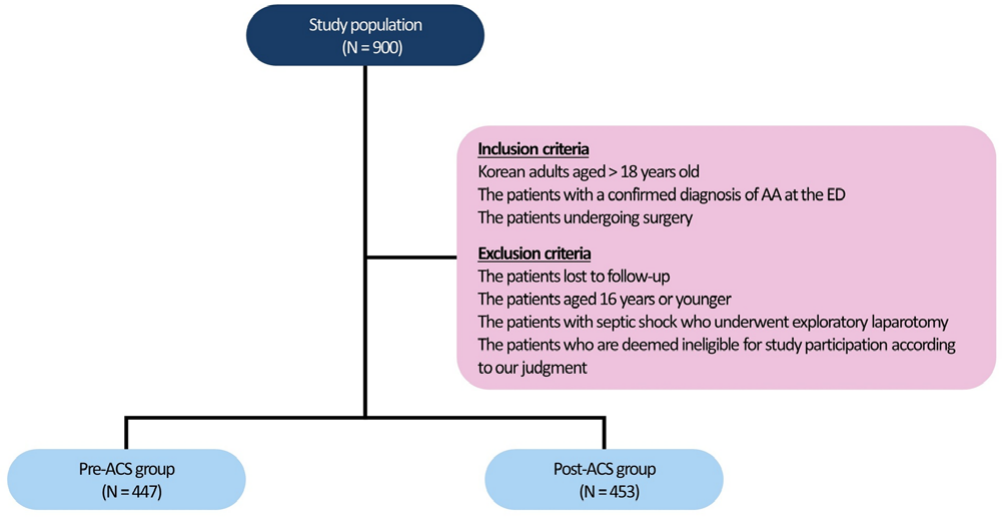
Study flow chart.

A total of 900 patients with AA were finally included in the current study, 447 and 453 of whom were divided into the pre-ACS group (n = 447) and the post-ACS group (n = 453), respectively (Fig. 2). Their baseline characteristics are represented in Table 1.

**Table 1:** Baseline characteristics of the patients (n = 900).

Variables	Values		*P* value
	Pre-ACS group (n = 447)	Post-ACS group (n = 453)	
Age (yr old)	52.1 ± 2.8 (34–64)	53.3 ± 3.6 (36–64)	.001^*^
Sex			.046^*^
** **Men	237 (53.0%)	210 (46.4%)	
** **Women	210 (47.0%)	243 (53.6%)	
BMI (kg/m^2^)	26.32 ± 6.15	26.71 ± 5.96	.186
Underlying diseases			
** **DM	20 (4.5%)	23 (5.1%)	.672
** **HTN	18 (4.1%)	18 (4.0%)	.967
Type of surgery			.001^*^
** **Open appendectomy	36 (8.1%)	21 (4.6%)	
** **Laparoscopic appendectomy	342 (76.5%)	396 (87.4%)	
** **Open extended appendectomy	15 (3.4%)	15 (3.3%)	
** **Laparoscopic extended appendectomy	54 (12.1%)	21 (4.6%)	
Conversion to open appendectomy	21 (4.7%)	24 (5.3%)	.680
SBP (mm Hg)	138.3 ± 39.4	146.1 ± 57.3	.001^*^
BT (°C)	37.2 ± 3.3	36.4 ± 4.3	.001^*^
WBC (10^6^/L)	11,420 (7780–14,080)	11,355 (8530–14,820)	.206
CRP (mg/dL)	2.79 (0.61–8.03)	2.80 (0.51–8.69)	.464
LA (mmol/L)	1.42 (1.01–1.87)	1.36 (0.95–1.80)	.001^*^

Values are the mean ± standard deviation or the number of the patients with percentage, where appropriate.

ACS = acute care surgery, BMI = body mass index, BT = body temperature, CRP = C-reactive protein, DM = diabetes mellitus, HTN = hypertension, LA = lactic acid, SBP = systolic blood pressure, WBC = white blood cells.

*Statistical significance at *P* < .05.

### 3.2. Key time intervals, outcomes, and LHS

**Figure 3.**
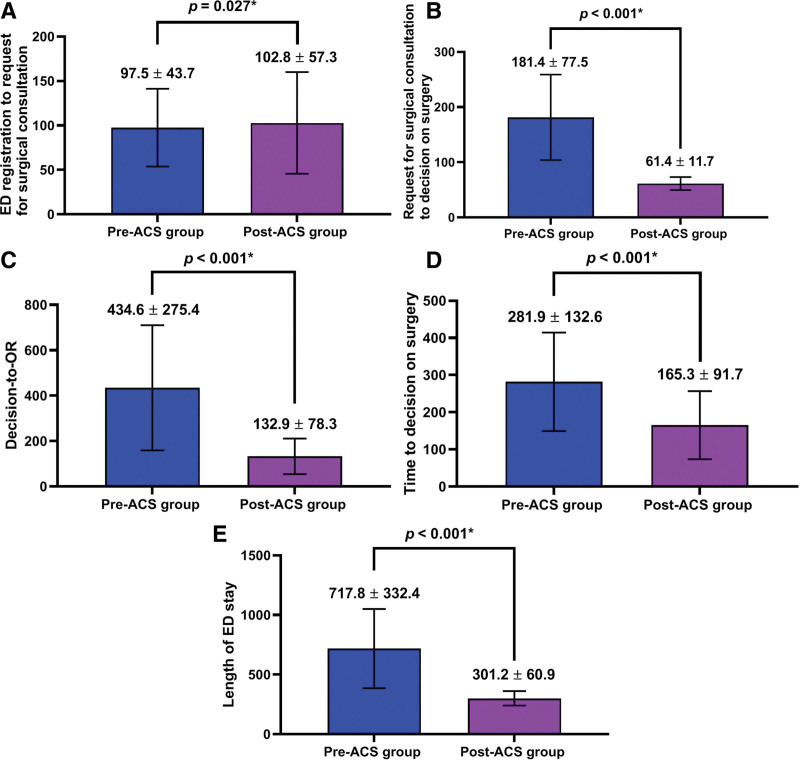
Key time intervals. (A) ED registration to request for surgical consultation, (B) request for surgical consultation to decision on surgery, (C) decision-to-operating room, (D) time to decision on surgery, and (E) length of emergency department stay. ED = emergency department.

**Figure 4.**
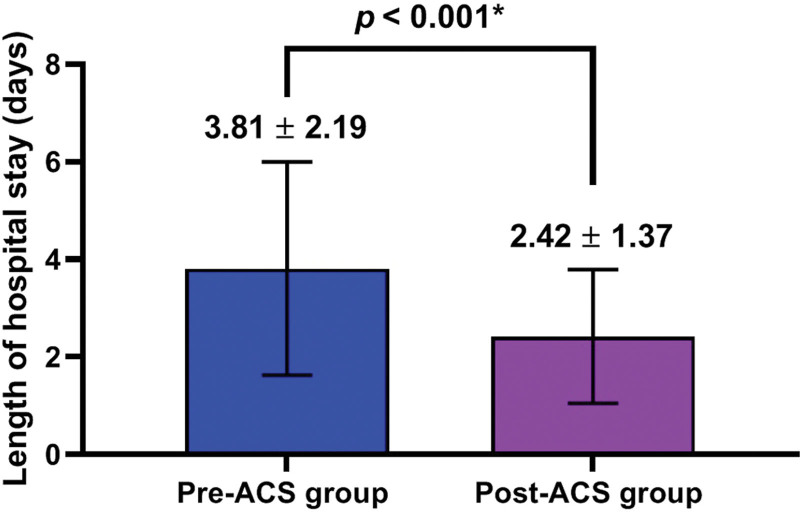
Length of hospital stay.

There were significant differences in key time intervals, outcomes and LHS between the 2 groups (*P* < .05) (Tables 2 and 3) (Figs. 3 and 4).

**Table 2:** Key time intervals (n = 900).

Variables	Values		*P* value
	Pre-ACS group (n = 447)	Post-ACS group (n = 453)	
ED registration to request for surgical consultation	97.5 ± 43.7	102.8 ± 57.3	.027*
Request for surgical consultation to decision on surgery	181.4 ± 77.5	61.4 ± 11.7	.001*
Decision-to-OR	434.6 ± 275.4	132.9 ± 78.3	.001*
Time to decision on surgery	281.9 ± 132.6	165.3 ± 91.7	.001*
Length of ED stay	717.8 ± 332.4	301.2 ± 60.9	.001*

Values are the mean ± standard deviation. The unit is min.

ACS = acute care surgery, ED = emergency department, OR = operating room.

* Statistical significance at *P* < .05.

**Table 3:** Outcomes and discharge within 24 or 48 hours.

Variables	Values		*P* value
	Pre-ACS group (n = 447)	Post-ACS group (n = 453)	
Outcomes			
** **Perforation	80 (17.90%)	46 (10.15%)	.001*
** **Other complications	25 (5.59%)	29 (6.42%)	.609
Discharge within 24 or 48 h			
** **Discharge within 24 h	12 (2.68%)	200 (44.15%)	.001*
** **Discharge within 48 h	110 (24.61%)	342 (75.50%)	.001*

Values are the mean ± standard deviation or the number of the patients with percentage, where appropriate.

ACS = acute care surgery, ED = emergency department.

* Statistical significance at *P* < .05.

## 4. Discussion

In Korea, the need for the introduction of trauma centers was brought to public attention through the treatment of the victim of gunshot wounds in the 2011 ship hijacking incident. As a result, 17 hospitals were selected as regional trauma center operators, and currently, 12 regional trauma centers have been established and are in operation.^[18]^ Each regional trauma center initially received funding for facilities and equipment necessary for trauma patient care, and the salaries of trauma-specialized physicians are continuously supported by the government. The Trauma Business Unit differentiates center evaluations and budget support through regular center assessments to enhance the quality of trauma centers.^[19–21]^ Through the regional trauma center program, prompt and specialized treatment for severely injured trauma patients has become possible, and trauma systems within each region are gradually being established with regional trauma centers at the core. One notable characteristic is that the government fully covers the salaries of up to 23 trauma-specialized physicians per trauma center, ensuring the profitability of hospitals operating trauma centers. In return for this support, however, trauma-specialized physicians are restricted from providing medical care to nontrauma patients, focusing solely on trauma patient treatment activities. On the other hand, the system for nontrauma emergency surgery in Korea, excluding trauma surgery, is in the early stages of discussion. Recently, a special law was implemented to limit the working hours of residents to a maximum of 80 hours per week, which has led to an increase in staffing shortages for nighttime emergency surgery and the management of surgical intensive care patients. In practice, nontrauma emergency surgery has mostly been performed in hospitals with general surgery departments as overnight on-call duty.^[10]^

In Korea, trauma center initiatives began in 2008, and currently, it is in its early stages. Since the early days of trauma center initiatives, the need for ACS has been recognized. Due to government regulations, however, trauma centers have been unable to implement ACS. Nevertheless, hospitals other than trauma centers are starting and establishing ACS, led by surgeons specializing in critical care medicine.^[10]^ Previous studies have reported that the ACS model was effective in improving LHS and postoperative outcomes in patients with AA.^[18–20]^

It remains a global concern that there is a decrease in the number of capable general surgeons despite the increased need for efficient management of general surgical emergencies.^[22,23]^ Due to heavy workload, relatively lower payment and a higher risk of legal problems, however, medical students and residents are decreasingly interest in becoming trauma surgeons in Korea. Moreover, the quality of service and patient satisfaction in the ED currently pose a challenge in a clinical setting. The ACS model established by the AAST can therefore provide a solution to such problems.^[24]^ Its benefits have been well described in the literature.^[25–27]^

ACS is a field of surgery that encompasses the treatment of trauma, critical care and nontrauma emergencies. It emerged in the early 2000s in the United States as a response to the crisis in trauma care and the lack of emergency surgical services. Due to various reasons, there was a decrease in the availability of trauma surgeons and a decline in the interest of specialized surgeons in emergency surgery, resulting in anticipated gaps in trauma and nontrauma emergency surgery. To address these issues, the concept of ACS was introduced, and it has now become a developing field worldwide.^[10]^

Positive effects of the ACS model have been advocated by several previous studies. According to a study investigating the educational effects of the ACS model in residents, it was described that they were provided with ample opportunities to encounter various nonemergency surgeries, allowing them to gain experience in decision-making and surgical techniques for complex surgical procedures.^[28]^ Moreover, the ACS model was reported to provide opportunities for maintaining professors’ emergency surgical skills, and residents could receive training through the optimal combination of regular and emergency surgeries.^[28]^ The ACS model not only had educational benefits but also had a positive impact on actual hospital revenue. In more detail, while the ACS model has been mainly responsible for performing emergency surgeries, general surgeons could increasingly perform regular surgeries. This has led to an increase in revenue on both sides.^[29]^ However, there were concerns that treating nontrauma emergency patients would increase the burden of treatment and surgery, potentially negatively affecting the outcomes of trauma patient care. Nevertheless, after the implementation of the ACS model, although surgical opportunities increased, the results of trauma patient care did not deteriorate.^[30,31]^ Additionally, it was found that surgeons on duty showed a higher level of satisfaction in the presence of the ACS model.^[32]^

To summarize, our results showed that there were significant differences in key time intervals, outcomes and LHS between the 2 groups. These results indicate that the implementation of the ACS model was effective in improving key time intervals, rates of perforation and discharge within 24 or 48 hours.

But our results cannot be generalized because there are 2 limitations of the current study: first, this is a single-center study. Therefore, the possibility of selection bias could not be completely ruled out. Second, some baseline characteristics showing significant differences between the 2 groups may serve as confounding variables. Further multi-center studies adjusting for such baseline characteristics are therefore warranted to corroborate our results.

## 5. Conclusion

In conclusion, our results showed that the implementation of the ACS model was effective in improving key time intervals, rates of perforation and discharge within 24 or 48 hours in the patients with AA.

## Author contributions

**Conceptualization:** Kyoung Won Yoon, Kyoungjin Choi.

**Formal analysis:** Kyoung Won Yoon.

**Writing – original draft:** Kyoung Won Yoon, Kyoungjin Choi, Donghyoun Lee.

**Software:** Kyoungjin Choi.

**Data curation:** Eunmi Gil, Chi-Min Park, Keesang Yoo.

**Writing – review & editing:** Donghyoun Lee.
